# Real-time tracking of the Bragg peak during proton therapy via 3D protoacoustic Imaging in a clinical scenario

**DOI:** 10.1038/s44303-024-00039-x

**Published:** 2024-09-17

**Authors:** Siqi Wang, Gilberto Gonzalez, Leshan Sun, Yifei Xu, Prabodh Pandey, Yong Chen, Shawn Liangzhong Xiang

**Affiliations:** 1 The Department of Biomedical Engineering, University of California, Irvine, CA 92617 USA; 2 Department of Radiation Oncology, University of Oklahoma Health Sciences Center, Oklahoma City, OK 73104 USA; 3 Department of Radiological Sciences, University of California at Irvine, Irvine, CA 92697 USA; 4 Beckman Laser Institute & Medical Clinic, University of California, Irvine, Irvine, CA 92612 USA

**Keywords:** Biotechnology, Cancer, Oncology, Engineering, Physics

## Abstract

Proton radiotherapy favored over X-ray photon therapy due to its reduced radiation exposure to surrounding healthy tissues, is highly dependent on the accurate positioning of the Bragg peak. Existing methods like PET and prompt gamma imaging to localize Bragg peak face challenges of low precision and high complexity. Here we introduce a 3D protoacoustic imaging with a 2D matrix array of 256 ultrasound transducers compatible with 256 parallel data acquisition channels provides real-time imaging capability (up to 75 frames per second with 10 averages), achieving high precision (5 mm/5% Gamma index shows accuracy better than 95.73%) at depths of tens of centimeters. We have successfully implemented this method in liver treatment with 5 pencil beam scanning and in prostate cancer treatment on a human torso phantom using a clinical proton machine. This demonstrates its capability to accurately identify the Bragg peak in practical clinical scenarios. It paves the way for adaptive radiotherapy with real-time feedback, potentially revolutionizing radiotherapy by enabling closed-loop treatment for improved patient outcomes.

## Introduction

**Fig. 1:**
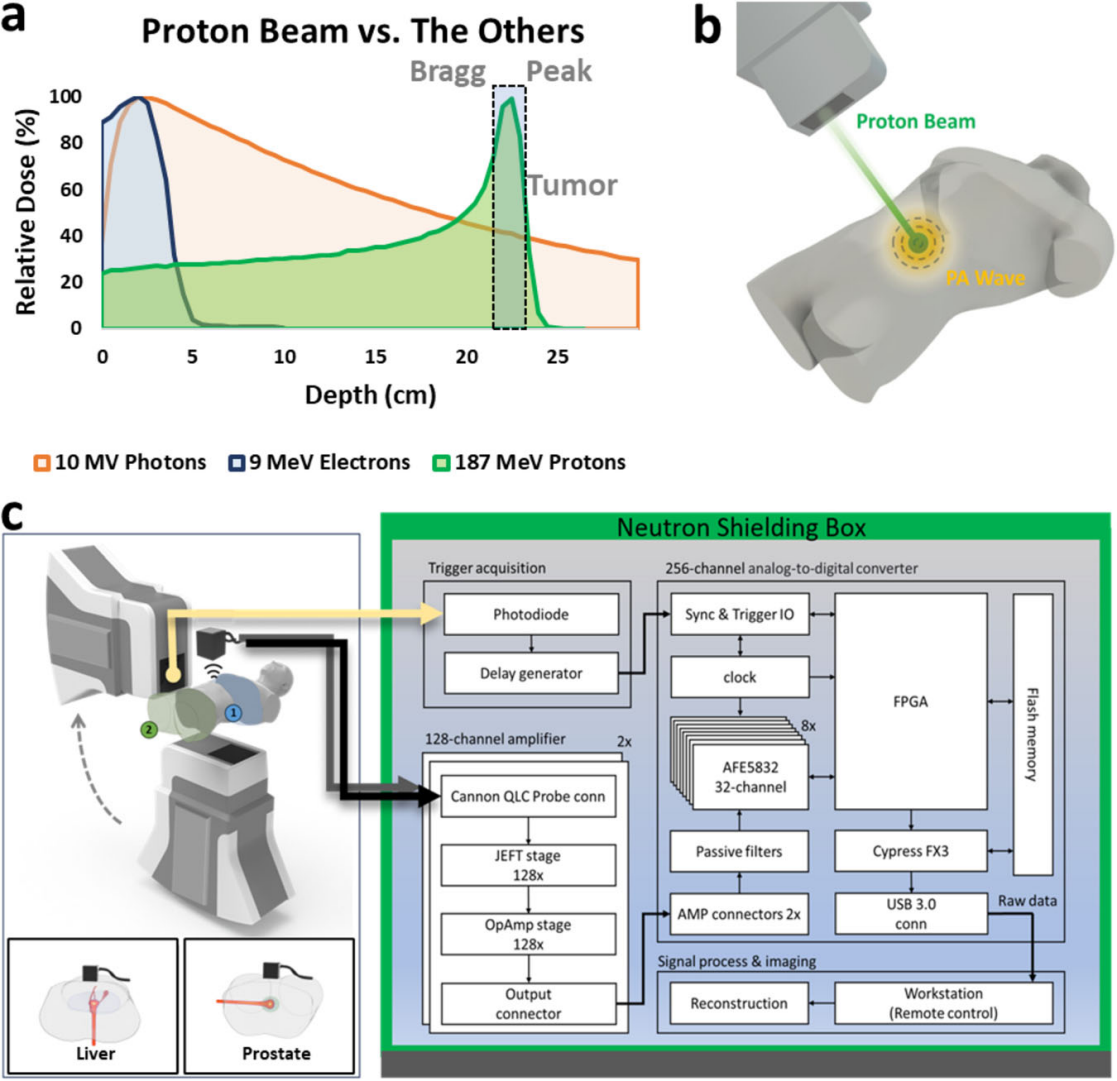
Schematic of protoacoustic imaging (PAI) for real-time tracking of Bragg peak during proton therapy. **a** Proton therapy takes advantage of the Bragg peak phenomenon, delivering radiation precisely to tumor sites, offering a superior alternative to conventional radiotherapy methods such as X-ray photons and electrons. **b** Illustration depicting the generation of protoacoustic (PA) waves by a proton beam in a clinical setting. **c** The PAI system employed in our experiment features a 256-element matrix ultrasound array. Each element is directly connected to a pre-amplifier channel and an ADC through 256 parallel channels. Data acquisition (DAQ) is synchronized with the trigger signal detected from the proton machine.

Cancer, a leading cause of death globally, is characterized by the rapid and uncontrollable division of its cells^1^. Radiation therapy, used in over half of cancer cases, effectively destroys or impedes cancer cells by damaging their DNA^2^. However, it adversely affects healthy cells, resulting in notable side effects and compromising the patient’s overall outcome^3^. Proton therapy leverages the distinct depth-dose characteristics of protons, known as the Bragg Peak (Fig. 1a), presenting a substantial benefit compared to conventional radiotherapy methods such as X-ray photon therapy and electron therapy^4^. This technique enables more precise tumor targeting while safeguarding adjacent sensitive organs, which may decrease side effects, heighten treatment efficacy, and boost patient quality of life^5^. By December 2021, an estimated 279,455 patients had received proton radiation therapy worldwide^6^. With over one hundred proton therapy centers established globally, the field is undergoing explosive growth^7^ (Supplementary Note 1).

The effectiveness of proton therapy, attributed to the precise location of the Bragg peak, faces challenges due to ‘range uncertainty'^8^. This uncertainty arises from factors like CT value conversion inaccuracies, changes in patient anatomy, and organ movement, making it difficult to pinpoint the exact location of the Bragg peak^9^. In its current form, radiotherapy operates as an open-loop treatment, meaning the radiation dose delivered to the patient isn’t directly verified^10^. This lack of direct feedback inherently reduces safety and accuracy. Therefore, accurately localizing the Bragg peak and providing real-time feedback is critical for improving the precision of radiotherapy. Techniques such as prompt gamma detection (PGD) and positron emitter tomography (PET) are developed to measure the proton beam range in vivo, yet face challenges in clinical accuracy^11^. Both methods also depend on sophisticated and large detector systems and provide only indirect information about the Bragg peak’s position^12^.

Protoacoustic imaging (PAI), or ionoacoustic tomography, is emerging as a viable alternative for Bragg peak localization in proton therapy, overcoming the limitations of PGD and PET^13–18^. While the experimental observation of acoustic waves induced by a proton beam during patient treatment was first reported in 1995^19^, there has been renewed interest in this technique over the last few years due to upgraded proton machines and ultrasound detectors^12,15,17,20–25^. Unlike PGD and PET, which require placing bulky and expensive gamma-ray detectors around patients, protoacoustic detection systems comprise a single detector or an array of ultrasound detectors that only require a small space and are more affordable^12,25–27^. Recent studies have utilized protoacoustics to reduce range uncertainty in proton therapy, employing various proton accelerators such as linacs^28^, synchrotrons^19,29^, cyclotrons^30,31^, and synchrocyclotron^14,32^, all showing promising outcomes. Initially, research predominantly involved single ultrasound transducers for basic point measurements^13,14,19,27^. Researchers initially explored the use of linear ultrasound arrays for obtaining 2D imaging of the Bragg peak^12,30^, but newer studies are increasingly focusing on more comprehensive 3D volumetric imaging of the Bragg peak, primarily through computational simulations^26,33,34^. A recent experiment employing a ring array ultrasound array with a 20 MeV proton beam from a Tandem accelerator represents a significant advancement in 3D protoacoustic imaging^17^. However, achieving 3D imaging capability requires mechanical rotation, and the lower energy level of this proton source is not compatible with the higher-energy proton machines (>100 MeV) used in clinical settings. A real-time tracking imaging system for monitoring the Bragg peak in 3D during proton therapy in a clinical scenario is still lacking.

In this article, we present an advanced 3D protoacoustic imaging (PAI) system utilizing a 2D matrix array of 16 × 16 elements, which significantly enhances the 3D volumetric imaging capability of localizing the Bragg peak during proton therapy (Fig. 1b). This technology is pivotal in enabling adaptive radiotherapy with real-time feedback, potentially revolutionizing proton therapy for human cancers, particularly in liver and prostate. The system’s real-time capabilities, with up to 75 frames per second, allow for accurate tracking of rapid pencil beam scanning during the treatment. It is also equipped with an integrated ultrasonic transducer, multichannel pre-amplifiers, and 256 parallel data acquisition channels, making it suitable for clinical environments. Its convenience, affordability, and compact design make it applicable to various therapies, including LINAC X-ray photon^35–48^, electron^49,50^, and FLASH radiotherapy^50–52^. Moreover, this PAI concept extends to other imaging technologies, for example, 3D ultrasonography^53^ and photoacoustic imaging^54–58^ offering broad clinical potential.

## Results and discussion

### 3D PAI system

Figure 1c shows the 3D PAI system’s schematic, comprising a clinical proton machine (Hyperscan S250i, Mevion, USA) for protoacoustic signal generation (Methods). Protoacoustic signals are captured by a 256-element matrix ultrasonic array (Doppler Tech Inc., Guangzhou, China), amplified, and processed by a custom 256-channel data acquisition system (Photosound Tech Inc., Houston, USA) (Supplementary Note 5). The trigger signal, detected by the combination of a photodiode and scintillator, has been used to synchronize the data acquisition process. Signals were processed and reconstructed using a 3D back-projection algorithm^59^. This configuration, which eliminates the need for mechanical scanning, enables real-time 3D Bragg peak imaging during proton therapy, achieving up to 75 frames per second with 10 averages at a repetition rate of 750 Hz from the proton machine.

### 3D visualization of Bragg peak

**Fig. 2:**
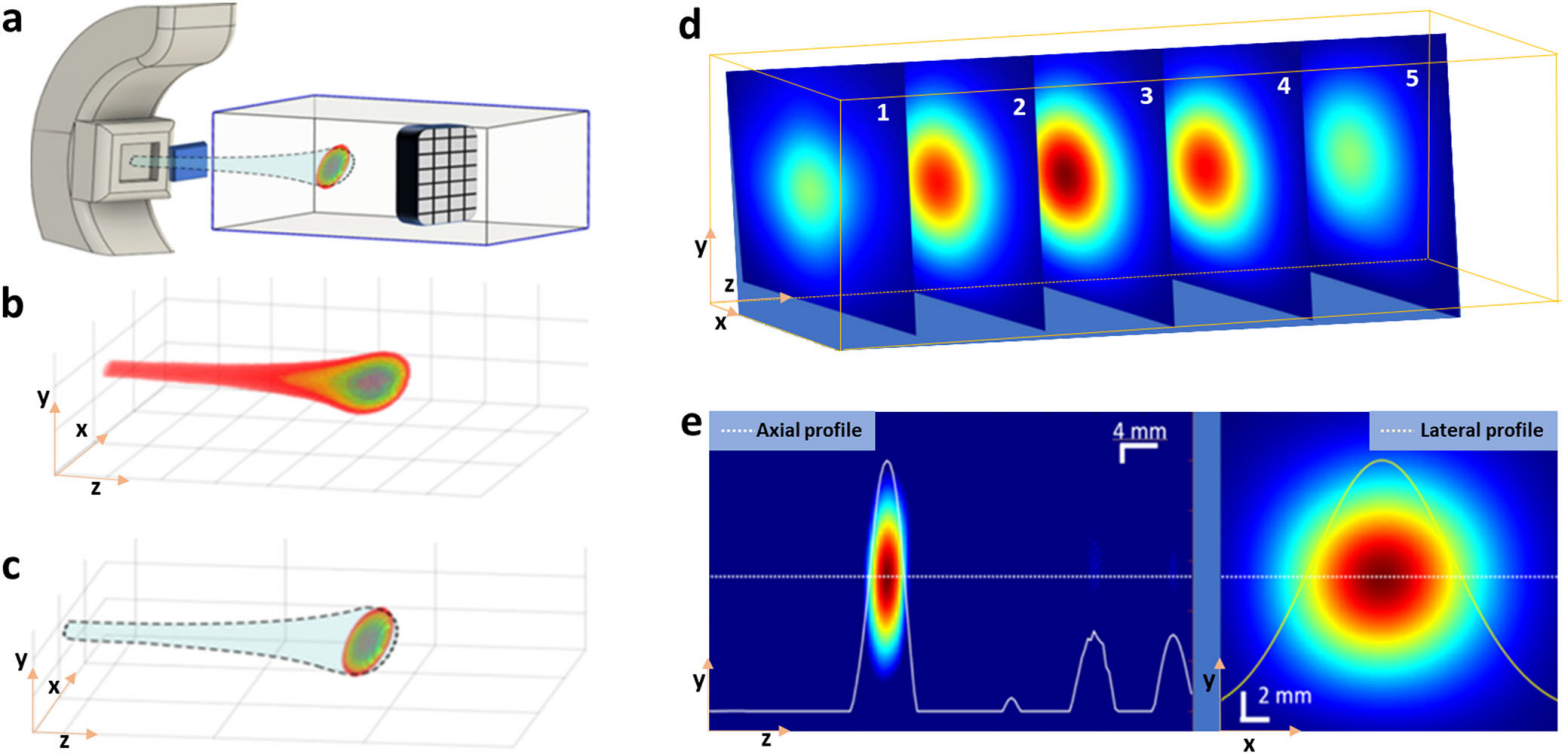
Three-dimensional (3D) visualization of the Bragg peak of a proton beam using protoacoustic imaging (PAI). **a** Orientation of the matrix array relative to the proton beam during our experiment. **b** Calculated proton dose distribution with TOPAS simulation in 3D. **c** 3D map depicting the Bragg peak obtained with PAI. **d** Five typical slices of PA images representing various cross-sections with 0.4 mm thickness of the Bragg peak in the X–Y plane of 108 MeV proton beam. **e** Protoacoustic (PA) image of the Bragg peak displayed in the Y–Z plane (left), and the PA image of the Bragg peak depicted in the X–Y plane (right).

The critical role of three-dimensional visualization of the Bragg peak within proton therapy for cancer treatment is profoundly significant. This peak signifies where the proton beam releases the maximum energy, making it pivotal for clinicians to target the tumor accurately with the highest dose of radiation while safeguarding the surrounding healthy tissues. This level of precision is crucial, especially for tumors located near or within essential structures. In the past, research in protoacoustics primarily focused on point measurements using a single transducer^14,16,22,29^ or on 2D imaging with a linear array^12,17,28,30,32^. Our study propels this field forward by utilizing a 2D matrix array composed of 256 (16 × 16) transducers positioned in front of the Bragg peak (Fig. 2a), thereby enabling the 3D volumetric imaging of the Bragg peak in proton therapy. With the 2D matrix array (size of 5 cm by 5 cm, with a center frequency of 1 MHz and a bandwidth of up to 60%) (Supplementary Note 2), we have successfully reconstructed a 3D PAI image. The 3D rendering of the Bragg peak at 108 MeV, captured through Protoacoustic Imaging (PAI) (Fig. 2c, Supplementary Video 1), aligns closely with the TOPAS simulation (Fig. 2b), showcasing its accuracy and potential for precise range measurements in proton therapy. This breakthrough is further evidenced by images in the X–Y plane and dose maps at various depths (Fig. 2d), offering a comprehensive view of the proton beam’s spatial distribution in water (Supplementary Video 2). Additionally, the visualization of two orthogonal planes crossing at the center of the Bragg peak (Fig. 2e) illustrates the precise location of the Bragg peak at the location of 30 mm (Fig. 2e, left), consistent with film measurement. The dose distribution map is displayed in the X–Y plane, as depicted in Fig. 2e (right). This novel imaging approach facilitates the 3D observation of the Bragg peak during proton therapy.

### Characterization of the protoacoustic imaging in 3D

**Fig. 3:**
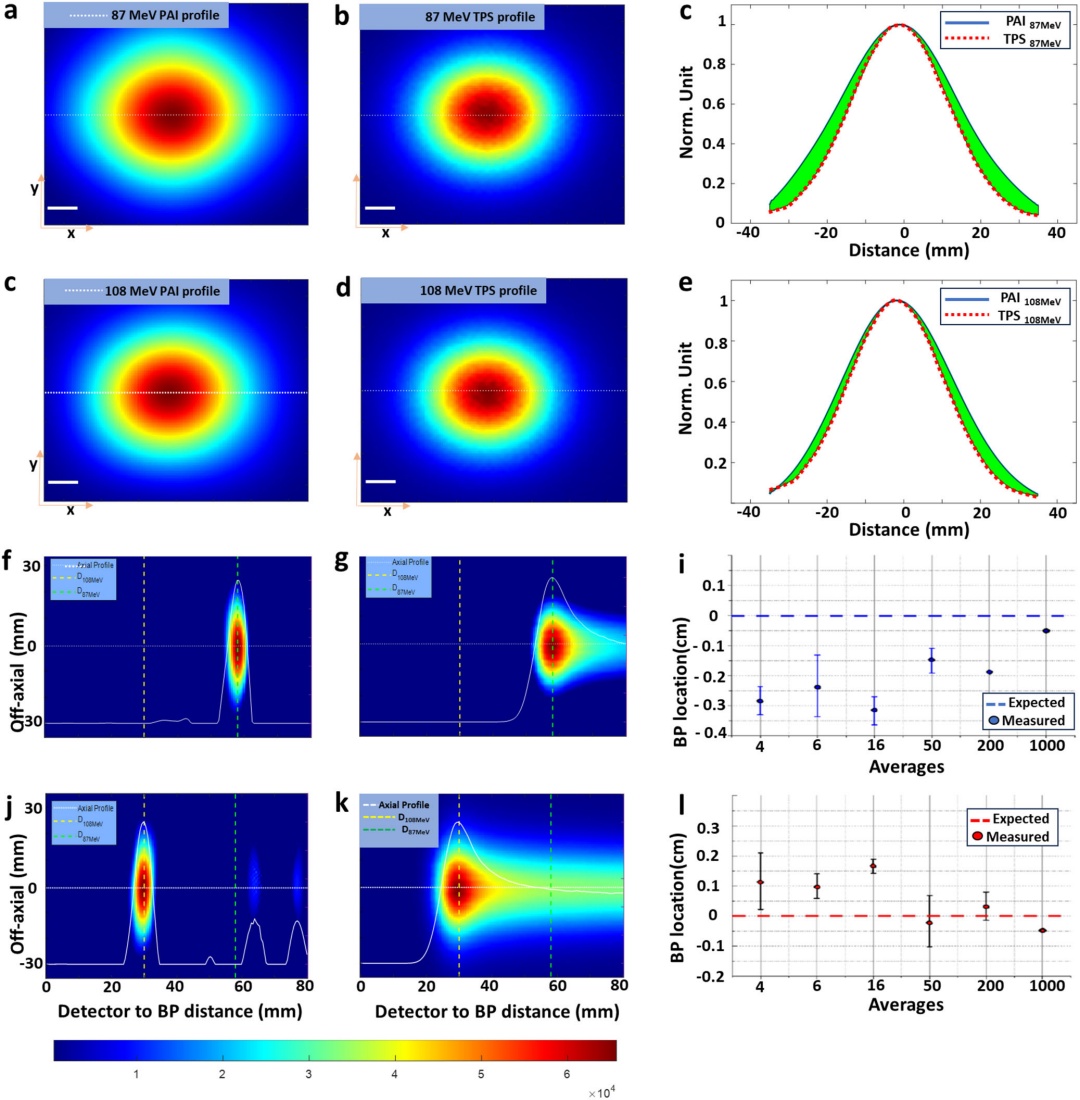
Characterization of protoacoustic imaging in 3D. **a** PA image of 87 MeV protons in the X–Y plane, **b** corresponding TOPAS simulation. **d** PA image of 108 MeV protons in the X–Y plane, **e** corresponding TOPAS simulation. **c**, **f** The profile comparison between the PAI and simulation along the dashed line in (**a**) and (**d**) correspondingly. The green color marks the difference between the two. **g**, **j** PA images of cross sections of the Bragg peak for 87 MeV and 108 MeV protons, respectively, with (**h**) and (**k**) showing the corresponding TOPAS simulations. **i** demonstrates PAI accuracy in localizing the Bragg peak to better than 2 mm with 50 more averages, while **l** exhibits PAI accuracy in localizing the Bragg peak to better than 1 mm with ~1000 averages.

To evaluate the precision of Protoacoustic Imaging (PAI) in identifying Bragg peaks, we conducted a comparison between the Bragg peak profiles reconstructed via PAI (Fig. 3a, d) and those predicted by an in-house TOPAS Monte Carlo simulation code (Fig. 3b, e)^60^. The reconstructed profiles exhibited Gaussian curves and sizes that closely matched those in the simulations (Fig. 3c, f). Figure 3g, j presents axial plane slices from PAI experiments for proton beams of 87 MeV and 108 MeV, respectively, with comparisons to corresponding simulation slices in Fig. 3h, k. The axial plane (X–Z plane) accuracy, crucial for verifying the range, showed that the reconstructed protoacoustic images aligned closely with the simulation predictions. The accuracy of the reconstructions improved with the number of averaging frames used, with 50 or more frames yielding deviations less than 2 mm from the planned positions for 87 MeV protons, as illustrated in Fig. 3i. This accuracy further improves, potentially reaching submillimeter levels, with an increased averaging number up to 1000 times, as suggested in Fig. 3j. Additionally, the imaging resolution of the PAI system was assessed through the line spread function, achieving ~1.4 mm resolution in the axial plan and ~3.2 mm resolution lateral plane which determined by element size of the ultrasound transducer, detailed in Supplementary Note 3. This examination underscores PAI’s capability for precise localization and visualization of Bragg peaks, offering significant promise for enhancing the accuracy of proton therapy treatments.

### Real-time tracking of Bragg peak

**Fig. 4:**
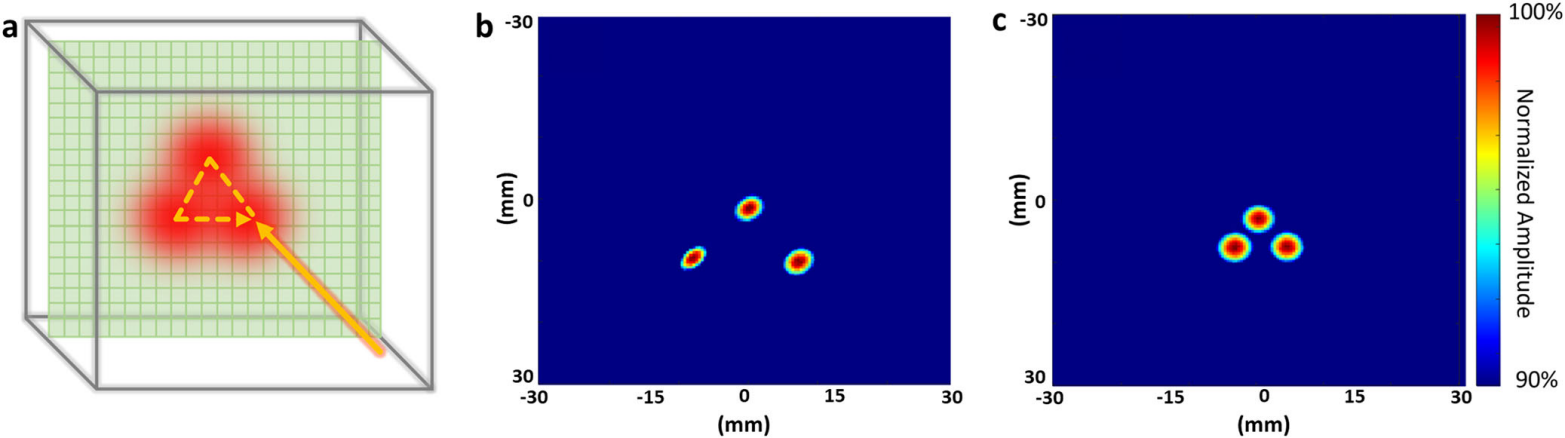
Real-time tracking of Bragg peak during proton pencil beam scanning. **a** Scanning pattern utilized in proton pencil beam scanning treatment. **b** Proton beam scanning demonstrating three different spots with a 10 mm step size (Supplementary Video 3). **c** Proton beam scanning demonstrates three different spots with a 5 mm step size (Supplementary Video 4).

PAI’s real-time 3D imaging capabilities can be utilized for the real-time monitoring of proton therapy, particularly in pencil beam scanning, as illustrated in Fig. 4a. In order to compare the actual spot positions with the theoretical value, three spots were scanned. The study utilized a 227 MeV proton beam, with the PAI detector array effectively monitoring the beam’s path with different step sizes (10 mm in Fig 4b vs. 5 mm in Fig. 4c). Reconstructions confirmed the designed patterns and spacings, affirming PAI’s accuracy and potential for real-time therapy monitoring and adaptive radiotherapy. Video recordings (Supplementary Videos 3 and 4) further demonstrate the real-time imaging capability (up to 75 frames per second with 10 averages), underscoring PAI’s versatility in monitoring proton therapy in clinical settings.

### 3D imaging of Bragg peak in liver treatment

**Fig. 5:**
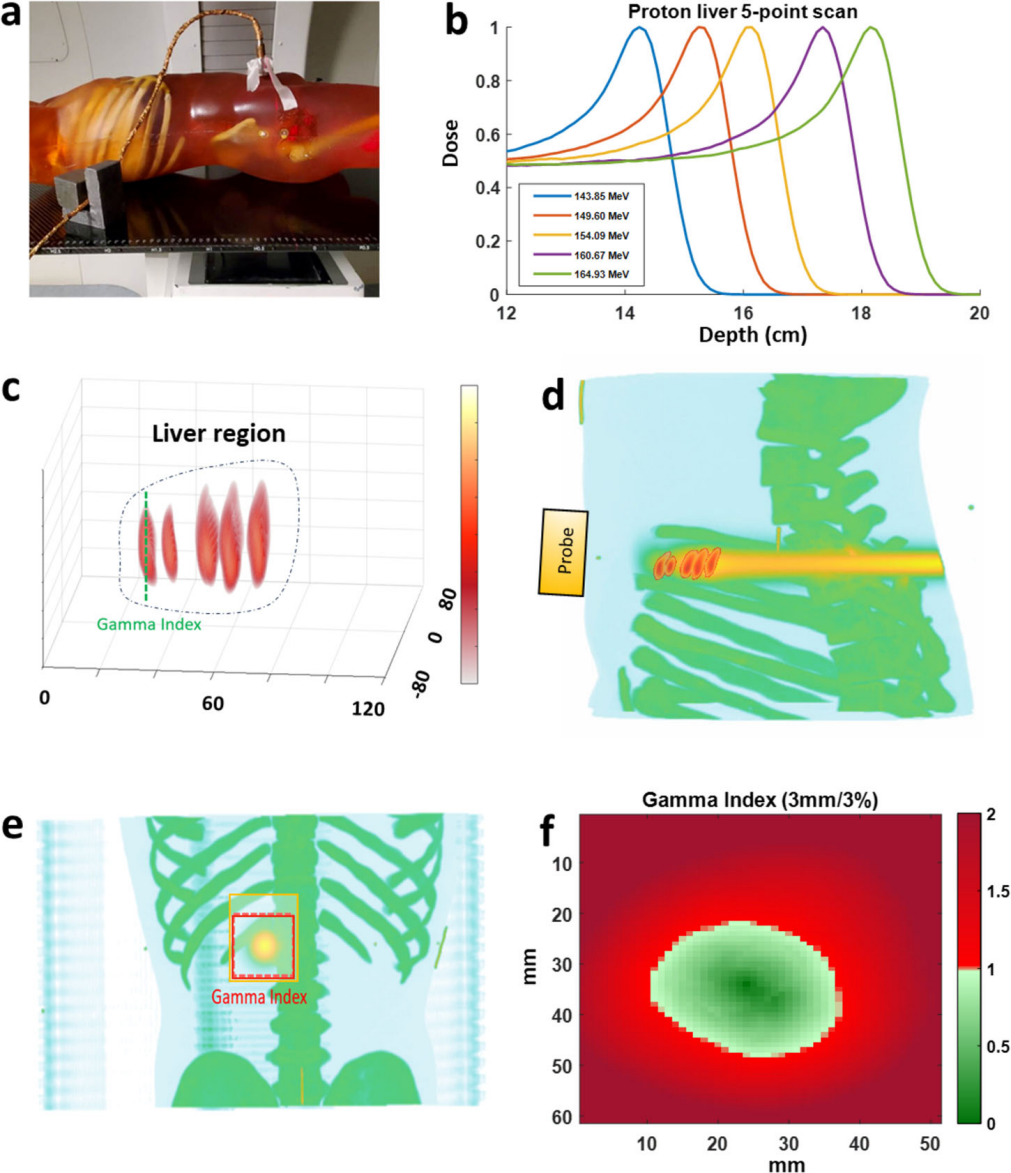
3D imaging of Bragg peak in liver treatment. **a** A photo capturing protoacoustic imaging in a clinical environment, where a matrix array is affixed to a human torso phantom during the experiment. **b** Graph depicting the dose profile of a proton scanning beam at varying energies. **c** Five 3D protoacoustic images showcasing Bragg peaks at different energies (143.85, 149.60, 154.09, 160.67, and 164.93 MeV). **d** Superimposition of the Bragg peak images onto the treatment plan in the X–Z plane using TOPAS. **e** Protoacoustic images of Bragg peaks overlaid onto planning CT scans in the X–Y plane. **f** Evaluation of gamma index (5 mm/5%) showing PAI's accuracy in Bragg peak localization, with over 95.73% precision.

To assess protoacoustic imaging (PAI) in localizing Bragg peaks in clinical scenarios, we utilized a liver cancer model within a human torso phantom, matching patient tissue properties in both planning CT and protoacoustic imaging (Supplementary Note 6). Treatment involved administering proton beams at five energy levels (143.85–164.93 MeV, Fig. 5b), directed from the patient’s back with an ultrasound probe on the abdomen (Fig. 5a**)**. PAI depicted relative dose delivery to the liver, revealing different beam locations in 3D imaging (Fig. 5c). Despite some axial distortion, PAI successfully resolved Bragg peak locations. In sagittal views, PAI images were overlaid with treatment planning dose distributions on the planning CT, as depicted in Fig. 5d. Comparing PAI measurements (Fig. 5e) of the Bragg peak of the proton beam to treatment plans (Fig. 5f, Supplementary Note 4) showed good alignment. In Fig. 5f, Gamma index mapping was employed to quantitatively assess PAI data against simulated doses, confirming the accuracy of PAI in dose mapping (Supplementary Note 7). The gamma index test, applying the 5 mm/5% criteria with a 10% dose threshold, reached an accuracy of 95.73%. This result signifies that 95.73% of the doses above 10% of the maximum measured dose aligned with the established ground truth. This level of accuracy is considered very high in the field of radiotherapy, demonstrating the effectiveness of the 5 mm/5% gamma index criteria. These findings underscore PAI’s potential in adaptive radiotherapy for precise Bragg peak localization.

### 3D imaging of Bragg peak in prostate treatment

**Fig. 6:**
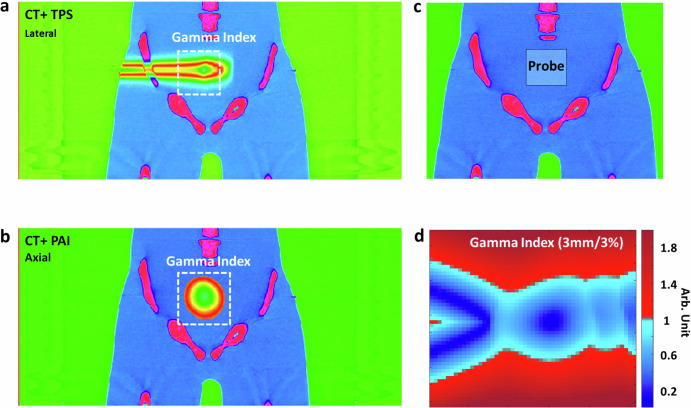
3D imaging of Bragg peak in prostate treatment. **a** Illustrates treatment planning for prostate proton therapy, featuring CT imaging overlaid with treatment planning dose distribution in the X–Z plane. **b** Presents a protoacoustic image superimposed onto the planning CT in the X–Y plane. **c** Depicts the positioning of the protoacoustic probe during the experiment. **d** Evaluation of the gamma index (5 mm/5%) demonstrates the precision of PAI in Bragg peak localization, achieving over 97.44% accuracy during prostate treatment.

We also showcased the effectiveness of PAI in monitoring prostate proton therapy (Fig. 6). Using the same human torso setup, we adjusted the proton beam gantry by 90 degrees to target the prostate laterally with a 171.1 MeV proton beam. The transabdominal ultrasound probe was employed to detect protoacoustic signals. Figure 6 illustrates the simulated dose distribution of proton therapy alongside PAI’s reconstructed distribution, focusing on a quintuple-point proton beam scan. Figure 6a presents the treatment planning for prostate proton therapy, integrating CT imaging with treatment planning dose in the X–Z plane. Figure 6b displays a protoacoustic image superimposed onto the planning CT in the X–Y plane. Figure 6c illustrates the positioning of the protoacoustic probe during the experiment. Figure 6d showcases the evaluation of the gamma index (3 mm/3%), demonstrating the precision of PAI in Bragg peak localization, achieving over 97.44% accuracy when applying the 5 mm/5% criteria with a 10% dose threshold, during prostate treatment. Real-time monitoring of the Bragg peak in vivo is crucial to ensure precise targeting of the prostate while minimizing radiation dose to adjacent critical organs, such as the rectum and bladder.

In summary, PAI showcases its potential in enhancing proton therapy by accurately localizing Bragg peaks in 3D. Its capability to offer detailed (5 mm/5% analysis achieving 95.73% accuracy) and real-time feedback (at 75 frames per second) is vital for verifying radiation delivery on the fly and enabling adaptive radiotherapy.

Our 3D protoacoustic imaging uses a 256-element 2D matrix array, marking the first 3D imaging of the Bragg peak with a clinical proton machine. This method improves upon traditional single detector or linear array for protoacoustic measurements^14,30^. It is particularly crucial for proton pencil beam scanning, where the precision of spot size, scanning position, and Bragg peak range greatly impacts dose distribution. Despite accurate correlation with treatment planning, some X–Z plane distortions (as shown in Fig. 3g, j) arise due to back projection algorithm limitations, common in ultrasound and photoacoustic imaging^59^. Future improvements could include advanced reconstruction algorithms, like model-based ^45,46,61^ or deep learning techniques^33,62,63^, to enhance imaging accuracy. Additionally, our dedicated pre-amplifier, data acquisition system, and wavelet filtering^64^ reduce the need for signal averaging, thereby enabling real-time imaging. When pulse-echo ultrasound is integrated with protoacoustic imaging, it can be utilized to manage tumor motion, with corrections made using interleaved ultrasound images^64,65^.

In 3D protoacoustic imaging, the lateral resolution is about 3.2 mm, influenced by the ultrasound element size in our matrix array (Supplementary Note 2). Axial resolution depends on proton pulse duration and the ultrasonic transducer’s detection bandwidth. We used a 1 MHz transducer with approximately 60% bandwidth and determined the imaging resolution with *R* = 0.88 *v*_*s*_/*f*_max_ around 1.01 mm resolution^66^. However, proton pulse durations of 4–20 µs reduce the axial resolution to 1.4 mm (Supplementary Note 3). Utilizing higher frequency transducers and narrower proton pulses could potentially allow for sub-millimeter resolution^17^, enhancing Bragg peak localization accuracy.

A key aspect of our study was testing our PAI imaging device in clinical scenarios, using a portable imaging system integrated with an ultrasound array and a parallel data acquisition system, suitable for clinical use. All experiments were performed using a clinical proton machine (S250i Proton Therapy System, Mevion, USA) with clinically relevant proton energies. We primarily conducted experiments on an adult human torso phantom designed for X-ray/CT and Ultrasound compatibility, replicating average adult male anatomy (Supplementary Note 6). This setup, ideal for proton treatment planning CT, allowed protoacoustic imaging tests without IRB protocols and prepared us for future patient testing and large clinical trials.

In conclusion, while the PAI system shows great promise in enhancing the precision and effectiveness of proton therapy, ongoing research, and development are essential to address its current limitations and fully realize its potential in clinical applications. The advancements in PAI technology and its integration into proton therapy could lead to more accurate, patient-specific treatments, ultimately improving outcomes for cancer patients undergoing proton therapy.

## Methods

### Experimental set-up

In the PAI system (shown Fig. 1c), there are five main components: (1) a rotating proton beam gantry with positions for prostate (180°) and liver (90°) procedures, (2) a pulse trigger signal from the S250i Proton Therapy System (Mevion, USA), (3) a 256-channel 2D ultrasound transducer array (Doppler Tech Inc., China), and (4) a custom 256-channel data acquisition system (Legion ADC, Photosound Tech Inc., USA). Acoustic waves from the proton beam are collected by the transducer array and digitized, enabling precise monitoring with (5) a computer. The proton source provides the proton beam with a pulse duration adjustable varies from 4 to 20 µs. The combination of a photodiode and a scintillator can detect the proton pulse and can be used as the trigger signal. Trigger signals are fed into a function generator (keysight, USA) and converted into digital pulses for the data acquisition system. For each trigger, approximately 75 µs of acoustic wave is collected with all 256 channels and converted into digital signals by the data acquisition system. The data will be transmitted to a computer and wait for postprocessing and image reconstruction.

### 2D matrix array

Matrix array transducers have been developed in this work for 3D imaging in real time. The matrix array transducers have 256 (16 × 16) active elements (3 mm in size and 0.2 mm in pitch) made of piezoelectric single crystals, PMN-PT, about 5 cm square (Supplementary Note 2). The matrix array, featuring a central frequency of 1 MHz and a fractional frequency bandwidth exceeding 60%, has undergone division into halves, with each half connected to 128-channel pre-amplifiers and a data acquisition system. This setup enables real-time 3D imaging capabilities.

### Pre-amplifiers and data acquisition

To address low signal amplitude in protoacoustic imaging with commercial ultrasound systems, each detector element is linked to a dedicated amplifier located nearby. This setup (Photosound Tech Inc., Houston, USA) includes 256 channel preamplifiers (40 dB) and secondary amplifiers (up to 51 dB) for SNR optimization, totaling a gain of up to 91 dB. This significantly reduces the number of averages needed for acquiring high-quality SNR protoacoustic signals (Supplementary Note 5).

### Protoacoustic imaging protocols

For our initial tests in visualizing the Bragg peak in 3D (Fig. 3), the matrix ultrasound array was immersed in a water tank which has been widely used in radiation therapy dosimetry measurements. A scintillator crystal was fixed in the 1-mm entrance window of the tank along the beam path to obtain trigger signals that initialize the data acquisition (DAQ) system. Individual spot energies with energies of 87 and 108 MeV were delivered to characterize the 3D protoacoustic images with an in-house TOPAS simulation. To showcase the real-time tracking of pencil beam scanning (Fig. 4), the same matrix ultrasound array was used, and three spot positions in the X–Y plane were specified. Proton beam scans were executed with two specific step sizes: 0.5 mm and 1 mm at 227 MeV beam energy. To evaluate PAI’s effectiveness with a human-like subject, clinical proton therapy setups were used with a human torso phantom (True Phantom Solutions, Ontario, Canada). IRB protocol is not required at this moment for phantom use. The phantom was CT scanned using a GE Lightspeed in a supine head-first position, following a clinical pelvis protocol with slice resolutions of 1.25 mm in the axial Z-direction and 1.02 mm in the XY-plane. Images were imported to the clinical treatment planning software Raystation ver. 12 (RaySearch Laboratories, Sweden) to calculate pencil beam doses for prostate (Fig. 5) and liver (Fig. 6). The phantom was positioned using skin markers and aligned with in-room lasers and kV-imagers. Raystation (version 12 A) calculated the dose for each treatment configuration. The dose grid was resized to match the physical dimensions of the CT image set, this ensured a matching overlay with protoacoustic imaging in MATLAB. The study considered two clinical scenarios: posterior-anterior beam delivery at 180° gantry for the liver and lateral beams at 90° for the prostate, with specified beam energies. Ultrasound gel ensured coupling between the array and the phantom. All experiments were performed with the synchrocyclotron’s Physics mode allowing the delivery of custom-made single spot plans. Clinical beam energies that reached the target depth were selected. The number of monitor units was set to an amount sufficiently large that the number of trigger events for protoacoustic signal acquisition would be met before the end of beam delivery.

### Human torso phantom

Our experiments extensively used a human-like phantom (True Phantom Solutions, Ontario, Canada), designed to resemble an average adult male’s anatomy. This highly detailed model is compatible with CT, and Ultrasound. We can simulate the planning CT with this human torso phantom. It includes realistic skeletal components, such as a complete spine and ribcage, and internal organs like the heart and liver. The phantom replicates human tissue properties with a sound velocity of 1400 ± 10 m/s, a density of 1.00 g/cm³, and an attenuation of 1.2 ± 0.2 dB/cm at 2.25 MHz. These features make it an ideal tool for accurate protoacoustic imaging studies (Supplementary Note 6).

### Signal processing and image reconstruction

Traditionally, the low signal-to-noise ratio (SNR) in protoacoustic signals requires multiple averages. This also reduces the frame rate, limiting real-time dose monitoring. To mitigate this, we employed a discrete wavelet transform (DWT) based filtering approach to denoise protoacoustic signals and minimize averaging^67^. Wavelet analysis via DWT extracts temporal and frequency data, allowing for selective filtering. Threshold coefficients from DWT analysis were synthesized back using inverse DWT (IDWT) with a sym8 wavelet and a 0.12-MHz cutoff frequency for the low-pass filter.

To reconstruct the protoacoustic image (PAI), the detected signals are “back projected” across the imaging space from the direction they were acquired. The protoacoustic pressure $p\left(r,\text{t}\right)$ detected at the transducer position ${\boldsymbol{r}}$ and time $t$ can be expressed by^68^:

1 $${\boldsymbol{p}}({\boldsymbol{r}},{\rm{t}})=\frac{1}{4\pi {{\boldsymbol{v}}}_{{\boldsymbol{s}}}^{2}}{\int }d{{\boldsymbol{r}}}^{\prime}\frac{1}{|{\boldsymbol{r}}-{{\boldsymbol{r}}}^{\prime}|}{{\rm{\eta }}}_{th}\rho \frac{\partial {D}_{{\boldsymbol{r}}}({{\boldsymbol{r}}}^{\prime},{t}^{\prime})}{\partial {t}^{\prime}}{|}_{{t}^{\prime}\,=\,t-\frac{|{\boldsymbol{r}}-{{\boldsymbol{r}}}^{\prime}|}{{{\boldsymbol{v}}}_{{\boldsymbol{s}}}}}$$

where ${\Gamma }$ is the Grüneisen parameter defined as: ${\Gamma }=\frac{{\boldsymbol{\beta }}{K}_{T}}{{C}_{{\boldsymbol{v}}}\rho }$. where *β* is the volumetric thermal expansion coefficient, and ${K}_{T}$ is the isothermal bulk modulus. Meanwhile, the initial pressure ${{\boldsymbol{p}}}_{0}\left({\boldsymbol{r}}\right)$ induced by radiation can be obtained by^69^:

2 $${{\boldsymbol{p}}}_{0}\left({\boldsymbol{r}}\right)=\Gamma {\eta }_{{th}}\rho {\text{D}}_{{\boldsymbol{p}}}({\boldsymbol{r}})$$

where ${D}_{{\boldsymbol{p}}}\left({\boldsymbol{r}}\right)={D}_{{\boldsymbol{r}}}\left({\boldsymbol{r}},t\right){\tau }_{{\boldsymbol{p}}}$ is the local energy deposition due to a single proton pulse with a pulse duration of ${\tau }_{{\boldsymbol{p}}}$. The pixel intensity in the PAI image, reconstructed from captured protoacoustic signals, represents the initial acoustic pressure. Therefore, the relative intensity image offers vital information regarding both the location of the proton beam and the amount of dose delivered to the target.

## Supplementary information

Supplementary Video 1

Supplementary Video 2

Supplementary Video 3

Supplementary Video 4

Supplementary information

## Data Availability

The data supporting the findings of this study are provided within the Article and its Supplementary Information. The raw and analyzed datasets generated during the study are available for research purposes from the corresponding authors on reasonable request.

## Code availability

The reconstruction code, the system-control software and the data-collection software are proprietary and used in licensed technologies, yet they are available from the corresponding author on reasonable request.

## References

[1] Brown, J. S. et al. Updating the definition of cancer. *Mol. Cancer Res.* **21**, 1142–1147 (2023). 37409952 10.1158/1541-7786.MCR-23-0411 PMC10618731

[2] Baskar, R., Lee, K. A., Yeo, R. & Yeoh, K.-W. Cancer and radiation therapy: current advances and future directions. *Int. J. Med. Sci.* **9**, 193–199 (2012). 22408567 10.7150/ijms.3635 PMC3298009

[3] Bentzen, S. M. Preventing or reducing late side effects of radiation therapy: radiobiology meets molecular pathology. *Nat. Rev. Cancer* **6**, 702–713 (2006). 16929324 10.1038/nrc1950

[4] Gray, P. J. & Efstathiou, J. A. Prostate cancer: proton therapy-revolutionary advance or diminishing returns? *Nat. Rev. Urol.* **10**, 128–129 (2013). 23399730 10.1038/nrurol.2013.16

[5] Durante, M., Orecchia, R. & Loeffler, J. S. Charged-particle therapy in cancer: clinical uses and future perspectives. *Nat. Rev. Clin. Oncol.* **14**, 483–495 (2017). 28290489 10.1038/nrclinonc.2017.30

[6] Particle Therapy Co-Operative Group (PTCOG). Patient statistics (2022). https://www.ptcog.ch/images/patientstatistics/Patientstatistics-updateDec2021_Sep62022_provisional.pdf (Accessed December 12, 2022).

[7] Chen, Z., Dominello, M. M., Joiner, M. C. & Burmeister, J. W. Proton versus photon radiation therapy: a clinical review. *Front. Oncol.* **13**, 1133909 (2023). 37064131 10.3389/fonc.2023.1133909 PMC10091462

[8] Lomax, A. J. Myths and realities of range uncertainty. *Br. J. Radiol.* **93**, 20190582 (2020). 31778317 10.1259/bjr.20190582 PMC7066970

[9] Paganetti, H. Range uncertainties in proton therapy and the role of Monte Carlo simulations. *Phys. Med. Biol.* **57**, R99–R117 (2012). 22571913 10.1088/0031-9155/57/11/R99 PMC3374500

[10] Lyu, Q., Neph, R. & Sheng, K. Tomographic detection of photon pairs produced from high-energy X-rays for the monitoring of radiotherapy dosing. *Nat. Biomed. Eng.* **7**, 323–334 (2023). 36280738 10.1038/s41551-022-00953-8 PMC10038801

[11] Parodi, K. & Polf, J. C. In vivo range verification in particle therapy. *Med. Phys.* **45**, e1036–e1050 (2018). 30421803 10.1002/mp.12960 PMC6262833

[12] Lascaud, J. et al. Investigating the accuracy of co-registered ionoacoustic and ultrasound images in pulsed proton beams. *Phys. Med. Biol*. **66**, 185007 (2021). 10.1088/1361-6560/ac215e 34438378

[13] Ahmad, M., Xiang, L., Yousefi, S. & Xing, L. Theoretical detection threshold of the proton-acoustic range verification technique. *Med. Phys.* **42**, 5735–5744 (2015). 26429247 10.1118/1.4929939 PMC4567582

[14] Caron, J. et al. Single pulse protoacoustic range verification using a clinical synchrocyclotron. *Phys. Med. Biol.* **68**, 045011 (2023). 10.1088/1361-6560/acb2ae PMC10567060 36634371

[15] Mast, T. D., Johnstone, D. A., Dumoulin, C. L., Lamba, M. A. & Patch, S. K. Reconstruction of thermoacoustic emission sources induced by proton irradiation using numerical time reversal. *Phys. Med. Biol*. **68**, 025003 (2023). 10.1088/1361-6560/acabfc PMC9976196 36595327

[16] Nie, W. et al. Proton range verification in homogeneous materials through acoustic measurements. *Phys. Med. Biol.* **63**, 025036 (2018). 29160776 10.1088/1361-6560/aa9c1f PMC5845813

[17] Kellnberger, S. et al. Ionoacoustic tomography of the proton Bragg peak in combination with ultrasound and optoacoustic imaging. *Sci. Rep.* **6**, 29305 (2016). 27384505 10.1038/srep29305 PMC4935843

[18] Lascaud, J. et al. Enhancement of the ionoacoustic effect through ultrasound and photoacoustic contrast agents. *Sci. Rep.* **11**, 2725 (2021). 33526802 10.1038/s41598-021-81964-4 PMC7851171

[19] Hayakawa, Y. et al. Acoustic pulse generated in a patient during treatment by pulsed proton radiation beam. *Radiat. Oncol. Investig.* **3**, 42–45 (1995).

[20] Jiang, Z. et al. Radiation-induced acoustic signal denoising using a supervised deep learning framework for imaging and therapy monitoring. *Phys. Med. Biol.* **68**, 235010 (2023). 10.1088/1361-6560/ad0283 PMC11000456 37820684

[21] Schauer, J. et al. Proton beam range verification by means of ionoacoustic measurements at clinically relevant doses using a correlation-based evaluation. *Front. Oncol.* **12**, 925542 (2022). 36408153 10.3389/fonc.2022.925542 PMC9670173

[22] Takayanagi, T. et al. On-line range verification for proton beam therapy using spherical ionoacoustic waves with resonant frequency. *Sci. Rep.* **10**, 20385 (2020). 33230208 10.1038/s41598-020-77422-2 PMC7683547

[23] Freijo, C., Herraiz, J. L., Sanchez-Parcerisa, D. & Udias, J. M. Dictionary-based protoacoustic dose map imaging for proton range verification. *Photoacoustics* **21**, 100240 (2021). 33520652 10.1016/j.pacs.2021.100240 PMC7820918

[24] Jones, K. C. et al. Acoustic-based proton range verification in heterogeneous tissue: simulation studies. *Phys. Med. Biol.* **63**, 025018 (2018). 29176057 10.1088/1361-6560/aa9d16 PMC5815855

[25] Otero, J., Felis, I., Ardid, M. & Herrero, A. Acoustic localization of Bragg peak proton beams for hadrontherapy monitoring. *Sensors* **19**, 1971 (2019). 31035504 10.3390/s19091971 PMC6539756

[26] Samant, P., Trevisi, L. M., Chen, Y., Zwart, T. & Xiang, L. 3-D protoacoustic imaging through a planar ultrasound array: a simulation workflow. *IEEE Trans. Radiat. Plasma Med Sci.* **7**, 83–95 (2023). 37588600 10.1109/trpms.2022.3177236 PMC10427128

[27] Takayanagi, T. et al. A novel range-verification method using ionoacoustic wave generated from spherical gold markers for particle-beam therapy: a simulation study. *Sci. Rep.* **9**, 4011 (2019). 30850625 10.1038/s41598-019-38889-w PMC6408528

[28] Patch, S. K., Santiago-Gonzalez, D. & Mustapha, B. Thermoacoustic range verification in the presence of acoustic heterogeneity and soundspeed errors - Robustness relative to ultrasound image of underlying anatomy. *Med. Phys.* **46**, 318–327 (2019). 30362132 10.1002/mp.13256

[29] Tada, J., Hayakawa, Y., Hosono, K. & Inada, T. Time resolved properties of acoustic pulses generated in water and in soft tissue by pulsed proton beam irradiation. *Med. Phys.* **18**, 1100–1104 (1991). 1661368 10.1118/1.596618

[30] Patch, S. K. et al. Thermoacoustic range verification using a clinical ultrasound array provides perfectly co-registered overlay of the Bragg peak onto an ultrasound image. *Phys. Med. Biol.* **61**, 5621–5638 (2016). 27385261 10.1088/0031-9155/61/15/5621

[31] Jones, K. C. et al. Experimental observation of acoustic emissions generated by a pulsed proton beam from a hospital-based clinical cyclotron. *Med. Phys.* **42**, 7090–7097 (2015). 26632062 10.1118/1.4935865

[32] Patch, S. K. et al. Thermoacoustic range verification during pencil beam delivery of a clinical plan to an abdominal imaging phantom. *Radiother. Oncol.* **159**, 224–230 (2021). 33798611 10.1016/j.radonc.2021.03.027 PMC13175219

[33] Jiang, Z. et al. 3D in vivo dose verification in prostate proton therapy with deep learning-based proton-acoustic imaging. *Phys. Med. Biol*. **67**, 215012 (2022). 10.1088/1361-6560/ac9881 PMC9647484 36206745

[34] Yu, Y., Qi, P. & Peng, H. Feasibility study of 3D time-reversal reconstruction of proton-induced acoustic signals for dose verification in the head and the liver: a simulation study. *Med. Phys.* **48**, 4485–4497 (2021). 34120348 10.1002/mp.15046

[35] Tang, S. et al. X-ray-induced acoustic computed tomography with an ultrasound transducer ring-array. *Appl. Phys. Lett.* **110**, 103504 (2017).

[36] Tang, S. S., Ramseyer, C., Samant, P. & Xiang, L. Z. X-ray-induced acoustic computed tomography of concrete infrastructure. *Appl. Phys. Lett.* **112**, 063504 (2018).

[37] Tang, S., Yang, K., Chen, Y. & Xiang, L. X-ray-induced acoustic computed tomography for 3D breast imaging: a simulation study. *Med. Phys.* **45**, 1662–1672 (2018). 29479717 10.1002/mp.12829

[38] Yan, Y. & Xiang, S. L. X-ray-induced acoustic computed tomography and its applications in biomedicine. *J. Biomed. Opt.* **29**, S11510 (2024). 38144393 10.1117/1.JBO.29.S1.S11510 PMC10740376

[39] Robertson, E. et al. X-ray-induced acoustic computed tomography (XACT): initial experiment on bone sample. *IEEE Trans. Ultrason. Ferroelect. Freq. Contr.* **68**, 1073–1080 (2021). 10.1109/TUFFC.2020.3032779 PMC8274389 33085608

[40] Wang, S., Ivanov, V., Pandey, P. K. & Xiang, L. X-ray-induced acoustic computed tomography (XACT) imaging with single-shot nanosecond X-ray. *Appl. Phys. Lett.* **119**, 183702 (2021). 34776515 10.1063/5.0071911 PMC8566011

[41] Samant, P., Trevisi, L., Ji, X. & Xiang, L. X-ray induced acoustic computed tomography. *Photoacoustics* **19**, 100177 (2020). 32215251 10.1016/j.pacs.2020.100177 PMC7090367

[42] Xiang, L. et al. X-ray acoustic computed tomography with pulsed x-ray beam from a medical linear accelerator. *Med. Phys.* **40**, 010701 (2012). 10.1118/1.4771935 PMC3537718 23298069

[43] Sun, L. et al. Towards quantitative in vivo dosimetry using x-ray acoustic computed tomography. *Med. Phys.*10.1002/mp.16476 (2023). 10.1002/mp.16476 PMC10656364 37203253

[44] Gonzalez, G. et al. Single-pulse X-ray acoustic computed tomography image guided precision radiation therapy. *Adv Radiat Oncol.* **8**, 101239 (2022). 10.1016/j.adro.2023.101239 PMC10276220 37334315

[45] Pandey, P. K. et al. Ring artifacts removal in X-ray-induced acoustic computed tomography. *J. Innov. Opt. Health Sci.* **15**, 2250017 (2022). 38645738 10.1142/s1793545822500171 PMC11031265

[46] Pandey, P. K. et al. Model-based X-ray-induced acoustic computed tomography. *IEEE Trans. Ultrason. Ferroelect. Freq. Contr.* **68**, 3560–3569 (2021). 10.1109/TUFFC.2021.3098501 PMC8739265 34310297

[47] Xiang, L., Tang, S., Ahmad, M. & Xing, L. High resolution X-ray-induced acoustic tomography. *Sci. Rep.* **6**, 26118 (2016). 27189746 10.1038/srep26118 PMC4870558

[48] Li, Y. et al. 3-D X-ray-induced acoustic computed tomography with a spherical array: a simulation study on bone imaging. *IEEE Trans. Ultrason. Ferroelect. Freq. Contr.* **67**, 1613–1619 (2020). 10.1109/TUFFC.2020.2983732 PMC7394001 32286967

[49] Oraiqat, I. et al. An ionizing radiation acoustic imaging (iRAI) technique for real-time dosimetric measurements for FLASH radiotherapy. *Med. Phys.* **47**, 5090–5101 (2020). 32592212 10.1002/mp.14358 PMC7722001

[50] Ba Sunbul, N. H. et al. A simulation study of ionizing radiation acoustic imaging (iRAI) as a real-time dosimetric technique for ultra-high dose rate radiotherapy (UHDR-RT). *Med. Phys.* **48**, 6137–6151 (2021). 34431520 10.1002/mp.15188 PMC8943858

[51] Kim, K., Pandey, P. K., Gonzalez, G., Chen, Y. & Xiang, L. Simulation study of protoacoustics as a real-time in-line dosimetry tool for FLASH proton therapy. *Med. Phys.*10.1002/mp.16894 (2023). 10.1002/mp.16894 PMC11186976 38116792

[52] El Naqa, I., Pogue, B. W., Zhang, R., Oraiqat, I. & Parodi, K. Image guidance for FLASH radiotherapy. *Med. Phys.* **49**, 4109–4122 (2022). 35396707 10.1002/mp.15662 PMC9844128

[53] Gonçalves, L. F. et al. Applications of 2-dimensional matrix array for 3- and 4-dimensional examination of the fetus: a pictorial essay. *J. Ultrasound Med.* **25**, 745–755 (2006). 16731891 10.7863/jum.2006.25.6.745 PMC1513649

[54] Kim, W., Choi, W., Ahn, J., Lee, C. & Kim, C. Wide-field three-dimensional photoacoustic/ultrasound scanner using a two-dimensional matrix transducer array. *Opt. Lett.* **48**, 343–346 (2023). 36638453 10.1364/OL.475725

[55] Liu, S., Song, W., Liao, X., Kim, T. T.-H. & Zheng, Y. Development of a handheld volumetric photoacoustic imaging system with a central-holed 2D matrix aperture. *IEEE Trans. Biomed. Eng.* **67**, 2482–2489 (2020). 31902752 10.1109/TBME.2019.2963464

[56] Li, L. et al. Single-impulse panoramic photoacoustic computed tomography of small-animal whole-body dynamics at high spatiotemporal resolution. *Nat. Biomed. Eng.* **1**, 0071 (2017). 29333331 10.1038/s41551-017-0071 PMC5766044

[57] Lin, L. et al. Single-breath-hold photoacoustic computed tomography of the breast. *Nat. Commun.* **9**, 2352 (2018). 29907740 10.1038/s41467-018-04576-z PMC6003984

[58] Kuniyil Ajith Singh, M. & Xia, W. Portable and affordable light source-based photoacoustic tomography. *Sensors* **20**, 6173 (2020). 33138296 10.3390/s20216173 PMC7663770

[59] Xu, M. & Wang, L. V. Universal back-projection algorithm for photoacoustic computed tomography. *Phys. Rev. E* **71**, 016706 (2005). 10.1103/PhysRevE.71.016706 15697763

[60] Perl, J., Shin, J., Schumann, J., Faddegon, B. & Paganetti, H. TOPAS: an innovative proton Monte Carlo platform for research and clinical applications. *Med. Phys.* **39**, 6818–6837 (2012). 23127075 10.1118/1.4758060 PMC3493036

[61] Pandey, P. K., Wang, S., Sun, L., Xing, L. & Xiang, L. Model-based 3-D X-ray induced acoustic computerized tomography. *IEEE Trans. Radiat. Plasma Med. Sci.* **7**, 532–543 (2023). 38046375 10.1109/TRPMS.2023.3238017 PMC10691826

[62] Jiang, Z. et al. Radiation-induced acoustic signal denoising using a supervised deep learning framework for imaging and therapy monitoring. *Phys. Med. Biol*. **69**, 085007 (2023). 10.1088/1361-6560/ad0283 PMC11000456 37820684

[63] Lang, Y., Jiang, Z., Sun, L., Xiang, L. & Ren, L. Hybrid-supervised deep learning for domain transfer 3D protoacoustic image reconstruction. *Phys. Med. Biol*. **69**, 085007 (2023). 10.1088/1361-6560/ad3327 PMC11076107 38471184

[64] Fu, L. & Jokerst, J. Interleave-sampled photoacoustic imaging: a doubled and equivalent sampling rate for high-frequency imaging. *Opt. Lett.* **47**, 3503–3506 (2022). 35838713 10.1364/OL.464293 PMC10100578

[65] Erlöv, T. et al. Regional motion correction for in vivo photoacoustic imaging in humans using interleaved ultrasound images. *Biomed. Opt. Express* **12**, 3312–3322 (2021). 34221662 10.1364/BOE.421644 PMC8221956

[66] Wang, L. V. & Hu, S. Photoacoustic tomography: in vivo imaging from organelles to organs. *Science* **335**, 1458–1462 (2012). 22442475 10.1126/science.1216210 PMC3322413

[67] van Bergen, R et al. Discrete wavelet transformation for the sensitive detection of ultrashort radiation pulse with radiation-induced acoustics. *IEEE Trans. Radiat. Plasma Med. Sci.* **8**, 76–87 (2023). 10.1109/trpms.2023.3314339 PMC11364354 39220226

[68] Hickling, S. et al. Ionizing radiation‐induced acoustics for radiotherapy and diagnostic radiology applications. *Med. Phys.* **45**, e707–e721 (2018). 29679491 10.1002/mp.12929

[69] Zhang, W. et al. Dual-Modality X-Ray-Induced Radiation Acoustic and Ultrasound Imaging for Real-Time Monitoring of Radiotherapy. *BME Front.* **2020**, 9853609 (2020). 37849969 10.34133/2020/9853609 PMC10521688

