# Supplementary material for: Real-time tracking of the Bragg peak during proton therapy via 3D protoacoustic Imaging in a clinical scenario
Source: Npj Imaging. 2024 Sep 17;2:34. doi: 10.1038/s44303-024-00039-x (PMC11893450; doi:10.1038/s44303-024-00039-x)
Supplement: Supplementary file 5 — Supplementary information [file 44303_2024_39_MOESM5_ESM.pdf]

npj Imaging, 2024, supplementary

Article

Real-time Tracking of the Bragg Peak during Proton Therapy via 3D Protoacoustic Imaging in a Clinical Scenario

Siqi Wang [1] *, PhD; Gilberto Gonzalez[2] *; Leshan Sun[1]; Yifei Xu[1], Prabodh Pandey[3], PhD; Yong Chen[2], PhD; Shawn (Liangzhong) Xiang, PhD[1,3,4]

* These authors contributed equally to this work

[1]The Department of Biomedical Engineering, University of California, Irvine, CA 92617, USA

[2]Department of Radiation Oncology, University of Oklahoma Health Sciences Center, Oklahoma City, OK, 73104, USA

[3]Department of Radiological Sciences, University of California at Irvine, Irvine, CA 92697, USA

[4]Beckman Laser Institute & Medical Clinic, University of California, Irvine, Irvine, CA 92612, USA

Corresponding author: Shawn (Liangzhong) Xiang, PhD, E-mail: liangzhx@hs.uci.edu

Yong Chen, PhD, E-mail: yong-chen@ouhsc.edu

Supplementary Note 1 | Overview of proton therapy vs conventional radiotherapy

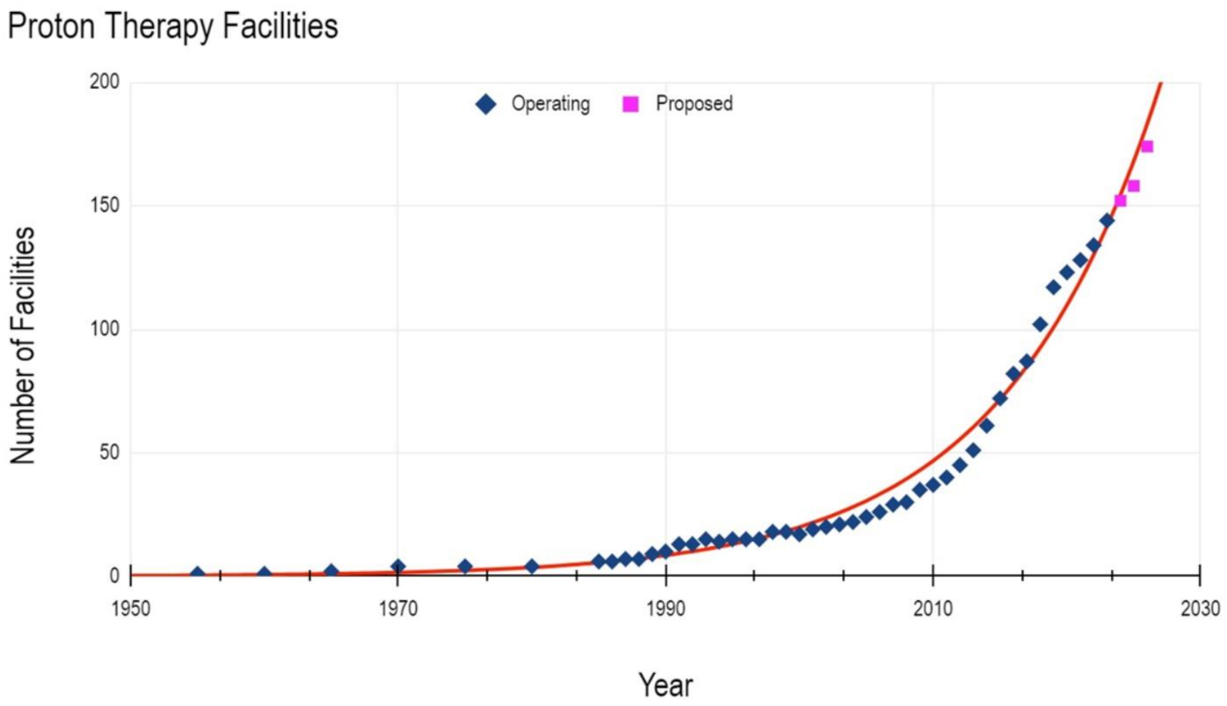

Supplementary Fig. S1: **Exponential grow in proton therapy for cancer patient**. As of October 2022, there were 118 operational proton radiation therapy centers globally, with 42 located in the United States. Additionally, 34 centers were under construction, and 32 were in the planning stage. (https://www.ptcog.ch/images/patientstatistics/Patientstatistics-update).

Radiation therapy is essential for treating cancer, aiding half of all patients in the US[1]. While photon therapy advances improve tumor targeting, elevated doses in nearby tissues hinder treatment escalation for some cancers. Proton therapy, leveraging the Bragg peak, promises significant enhancements in dose distribution, particularly benefiting pediatric and challenging-to-reach tumors[2]. For example, conventional 10 MV X-ray photon-based radiotherapy predominantly delivers the dose to the skin, with exponential decay as it reaches the tumor area. In contrast, a 9 MeV electron beam penetrates less than 5 cm into the patient, while a 187 MeV proton beam can reach depths of up to 24 cm, concentrating the majority of the radiation dose at the tumor site (shown in **Fig. 1a**).

Previously, proton centers were prohibitively expensive, with costs reaching up to $400 million. However, advancements in accelerator technology have drastically reduced the price to $30 million for a single-room proton machine. As of October 2022, there were 118 operational proton radiation therapy centers worldwide, including 42 in the United States, with 34 under construction and 32 in the planning stage. By December 2021, approximately 279,455 patients globally had received proton radiation therapy, indicating exponential growth in its clinical utilization. Therefore, our protoacoustic imaging-guided precision proton therapy has the potential to enhance the advantages of proton therapy over conventional radiotherapy.

Supplementary Note 2 | 2D matrix array and timing sequence utilized in protoacoustic imaging

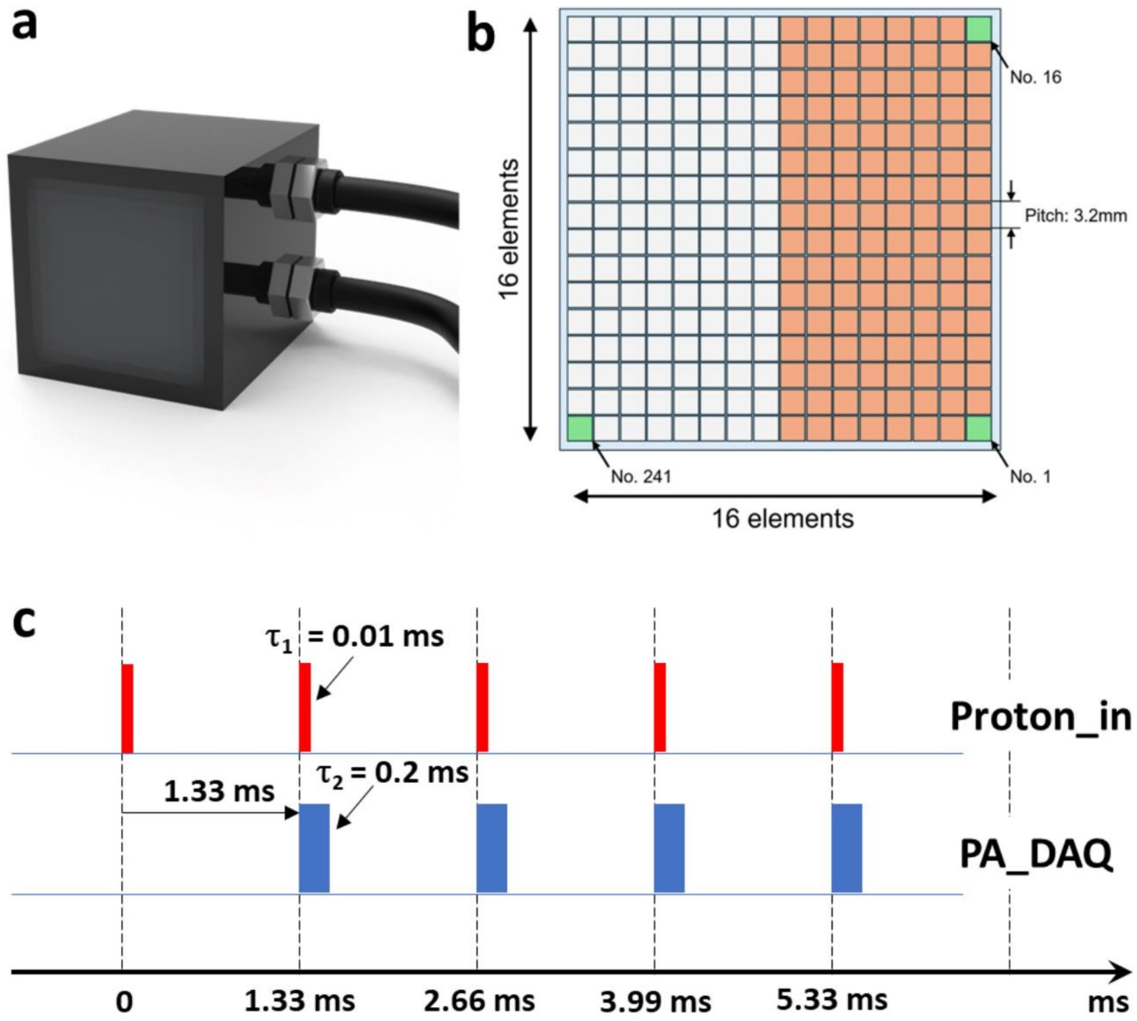

Supplementary Fig. S2: **Technique details in protoacoustic imaging**. (a) Photograph of the 2D matrix ultrasound array utilized in the experiment. (b) Schematic of the matrix array design comprising 256 elements with dimensions of 5 cm square. The individual element size and pitch measure 3 mm and 0.2 mm, respectively. (c) Timing sequence for real-time protoacoustic imaging. The timing diagram illustrates a PAI system with a 750 Hz trigger signal for proton excitation. PA signal acquisition is initiated with a 1.33 ms delay relative to proton beam firing, and approximately 75 microseconds are required to complete the protoacoustic data acquisition process.

Supplementary Note 3 | Imaging resolution for the PAI system

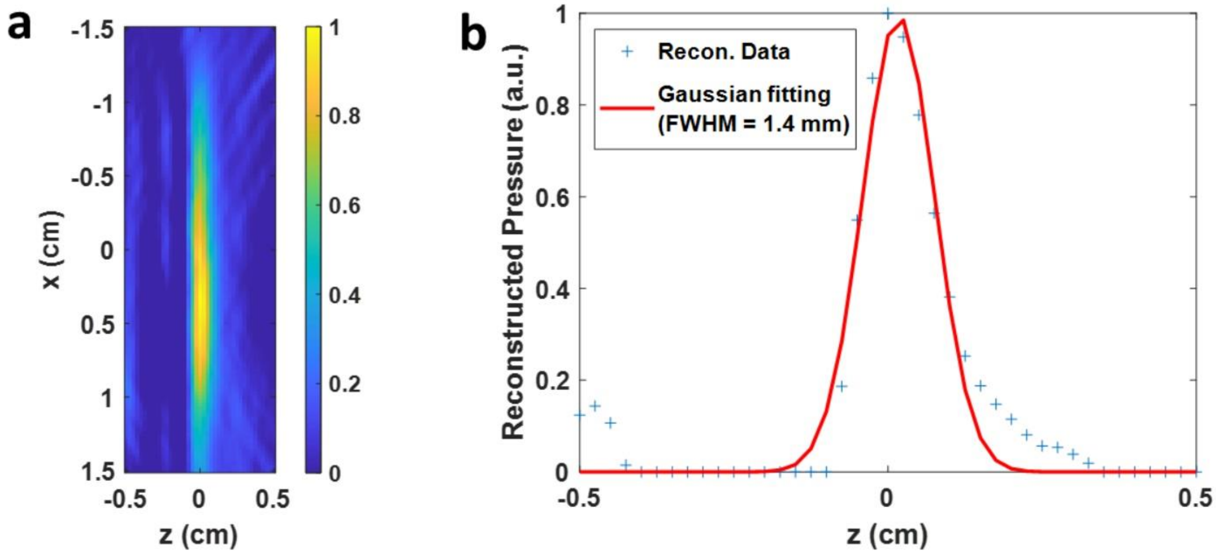

Supplementary Fig. S3: **The imaging resolution test for the matrix array system**. a) PAI imaging with a lead bar of 1 mm × 1.5 mm within the proton beam in the X-Z plane. b) Line spread function (LSF) of the PAI in the axial direction determines the axial resolution about 1.4 mm.

In **Fig. S3**, the PAI result illustrates a small piece of lead bar within the proton beam in a lateral plane. Fig. 3d presents the normalized intensity profile along x=0 mm line in **Fig. S3a**, where pixel intensities are represented by dots. The curve depicts the fitted line spread function, with a full-width at half-maximum of 1.4 mm, indicating an approximate lateral spatial resolution of 1.4 mm.

Supplementary Note 4 | TOPAS simulation for proton treatment planning

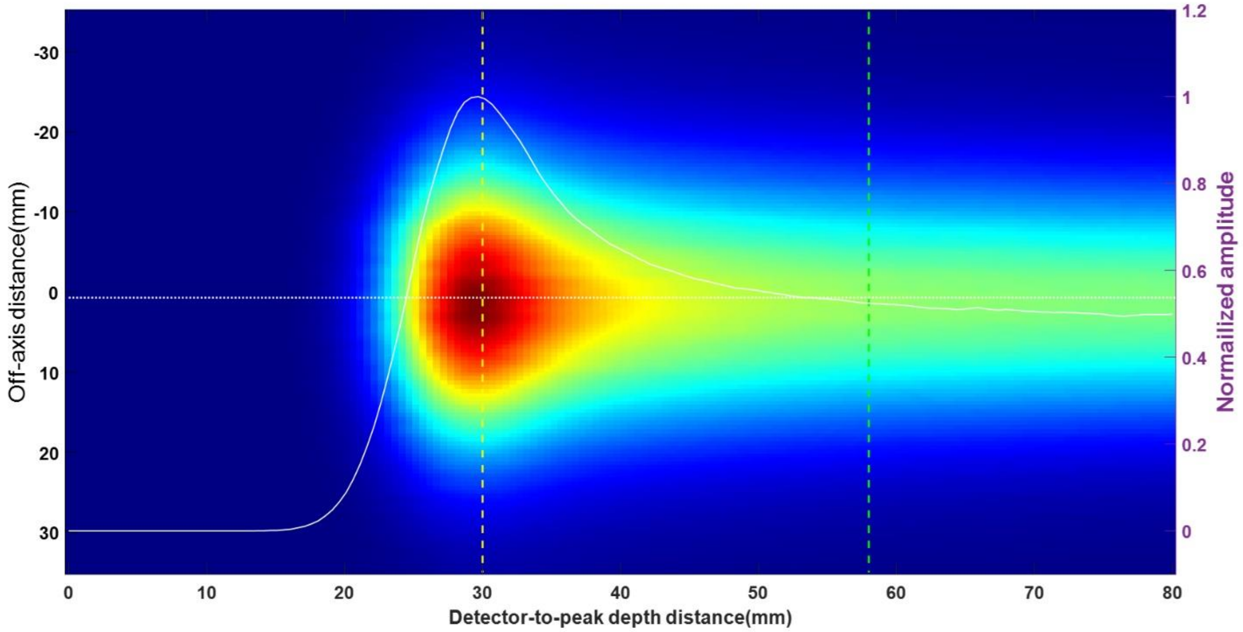

Supplementary Fig. S4: **TOPAS simulation for proton treatment planning**.

The study employed Monte Carlo toolkit TOPAS version 3.7 to simulate the beam delivery system. Developed as an extension of the GEANT4 10.1.p02 toolkit, TOPAS was specifically tailored for proton therapy, offering user-friendly features. It has been experimentally validated as an effective tool for replicating beam data from passive scattering proton systems.

The default modular physics list was applied across all simulations. This default physics list is tailored to simulate electromagnetic and nuclear interactions occurring within proton beams, specifically those up to 250 MeV in low-Z materials. The simulation parameters were based on an in-house model of the Mevion s250i synchrocyclotron, as detailed in a study that benchmarked the energy modulation system (EMS) and Adaptive Aperture (AA) with commissioning beam model data[3].

To collect the dose data, a large detector mesh as a parallel world in water material ensured a smooth PDD (Percentage Depth Dose), while a large bin-number ensured fine resolution in the z-direction to prevent distortion in high gradient areas. The depth of the Bragg peak was defined at the 90% dose in the distal fall-off (D90). The dose data was sent to MATLAB for analysis.

Supplementary Note 5 | Pre-amplifiers and data acquisition

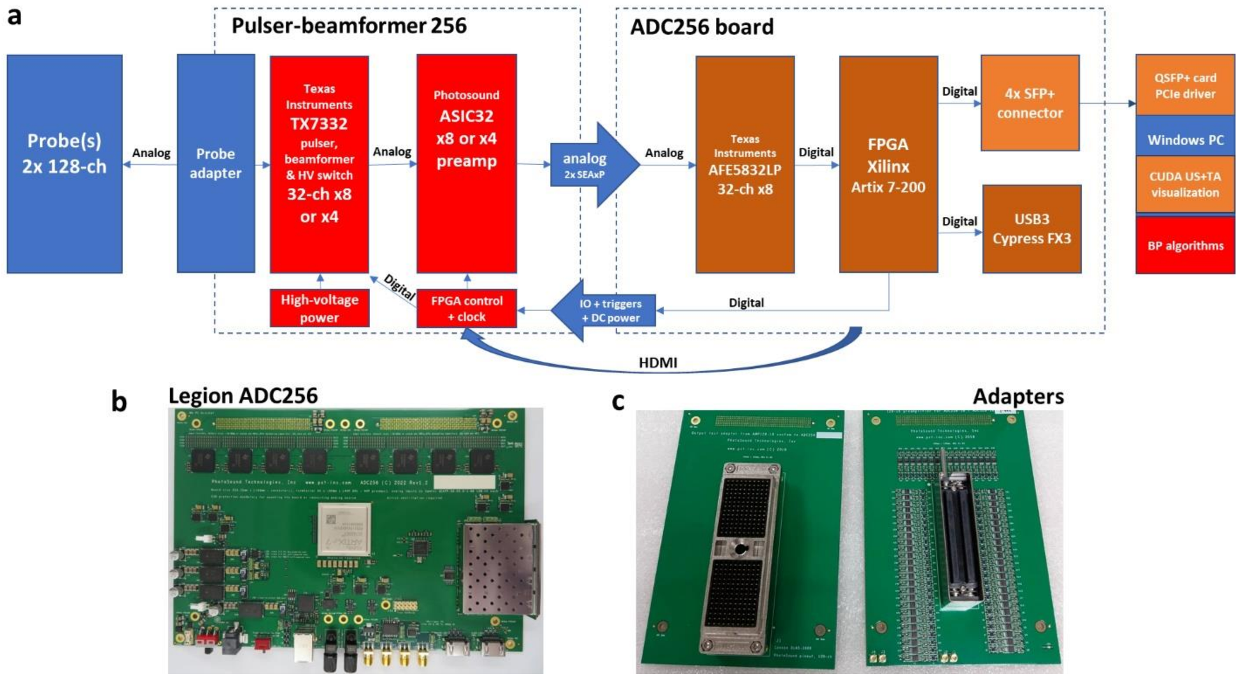

Supplementary Fig. S5: **Pre-amplifiers and data acquisition system.** (a) Schematic diagram shows how the protoacoustic signal being detected and amplified by dedicated preamplifiers and digitized by 256 parrel data acquisition channels. (b) The Legion ADC 256 board, and (c) The adapters for connecting the matrix array ultrasound probe and electronics system for preamplification and data acquisition.

Supplementary Note 6 | Human torso

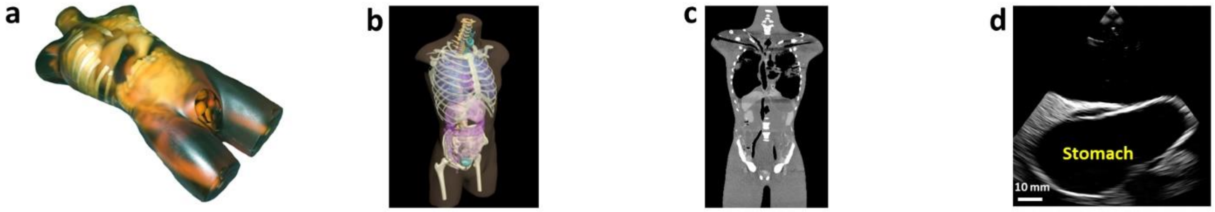

e

Type of Tissue	Sound Velocity [m/s]	Density $[g/cm^3]$	Attenuation @ 2.25MHz [dB/cm]	Hardness	HU Values
Organs (liver, kidneys, stomach, intestines, etc.)	1400 ± 10	0.99	1.0 ± 0.2	20	110
Body Tissue	1400 ± 10	1.00	1.2 ± 0.2	30	-25
Tumor Feature	1400 ± 10	1.01	1.2 ± 0.2	30	N/A
Cortical Bone	3000 ± 30	2.31	6.4 ± 0.3	N/A	1300
Trabecular Bone	2800 ± 50	2.03	21 ± 2	N/A	800
Thermal Conductivity (Bone)	Specific Heat Capacity (Bone)	Thermal Diffusivity (Bone)	Thermal Resistivity (Bone)	Specific Heat (Bone)	Speed of Sound (Bone)
0.776 W/ m K	1.040 MJ/ m^3 K	0.746 mm^2/ s	1.289 m K/ W	0.978 J/ g Deg Celsius	3070 m/s

Supplementary Fig.S6: **Technique details of the human torso used in the experiments.** (a) Photograph of the human torso and its frontal view design depicted in (b). (c) CT scans of the torso. (d) Ultrasound scan in the abdomen area. (e) Table listing the main parameters related to CT imaging and ultrasound imaging.

Supplementary Note 7 | Gamma index

a

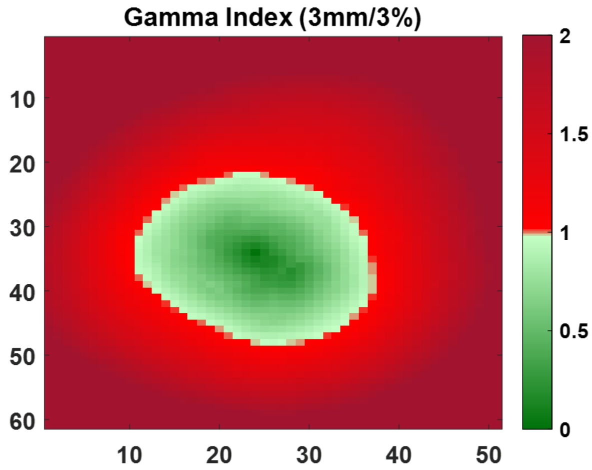

b

Gamma Index (Liver)	10% dose pass rate	50% dose pass rate
3mm/3%	38.58%	70.66%
3mm/5%	39.15%	71.71%
5mm/5%	95.73%	100.00%
**Gamma Index (Prostate)**	**10% dose pass rate**	**50% dose pass rate**
3mm/3%	61.34%	98.43%
3mm/5%	62.01%	98.76%
5mm/5%	97.44%	100.00%

Supplementary Fig. S7: **Details on calculating the Gamma index to assess the accuracy of PAI imaging against film measurements in clinical settings**. (a) An example for 3mm/3% Gamma Index analysis. (b) Table for the details in Gamma Index analysis for both liver and prostate cases.

The Gamma index assesses the agreement between planned and delivered dose distributions by comparing them and considering dose differences and distance-to-agreement criteria. The "3mm" criterion denotes the spatial distance-to-agreement, where points within 3 millimeters of each other are considered in agreement. The "3%" criterion signifies the dose difference tolerance, with points having dose differences less than 3% of the maximum dose considered in agreement.

To calculate the Gamma index, each point in the measured dose distribution is compared to the corresponding point in the planned dose distribution. If both dose difference and distance-to-agreement criteria are met, the point passes the Gamma test. The Gamma index is then calculated as the percentage of points passing the test relative to the total number of points compared. A Gamma index value below 1 indicates good agreement between planned and delivered dose distributions, while values above 1 suggest discrepancies that may require further investigation or adjustment of the treatment plan. The gamma index test, using the 5mm/5% criteria with a 10% dose threshold, achieved an accuracy of 95.73%. This indicates that 95.73% of the measured doses exceeding 10% of the maximum dose corresponded accurately to the predefined standard. It is observed that the accuracy decreases at lower gamma index thresholds, which is expected. This can be attributed to the use of the back projection algorithm and the inability to reconstruct the surrounding background with high precision.

Overall, the Gamma index offers a quantitative assessment of treatment plan accuracy, ensuring the quality and safety of radiation therapy treatments.

Supplementary video 1 | 3D visualization of the Bragg peak rotating at various angles

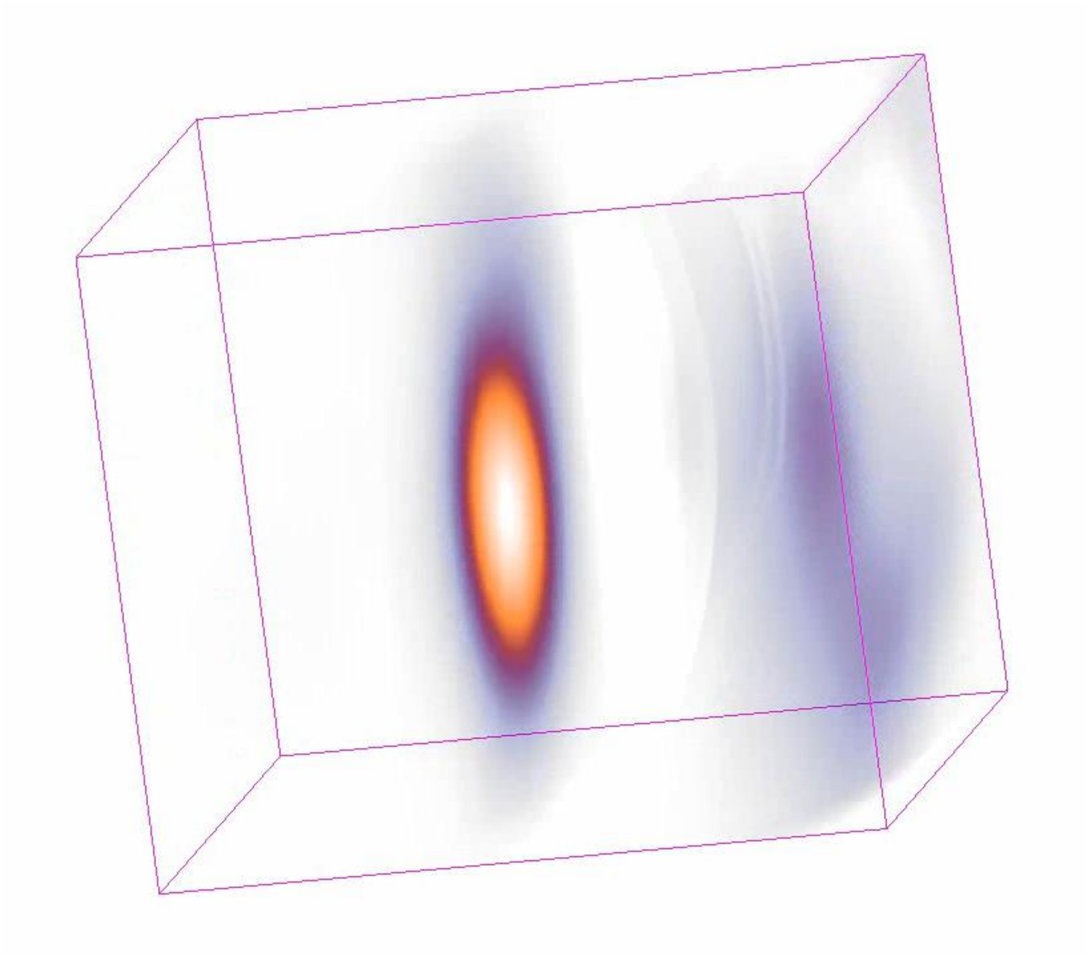

Supplementary video 2 | 3D visualization of the Bragg peak depicted in slices

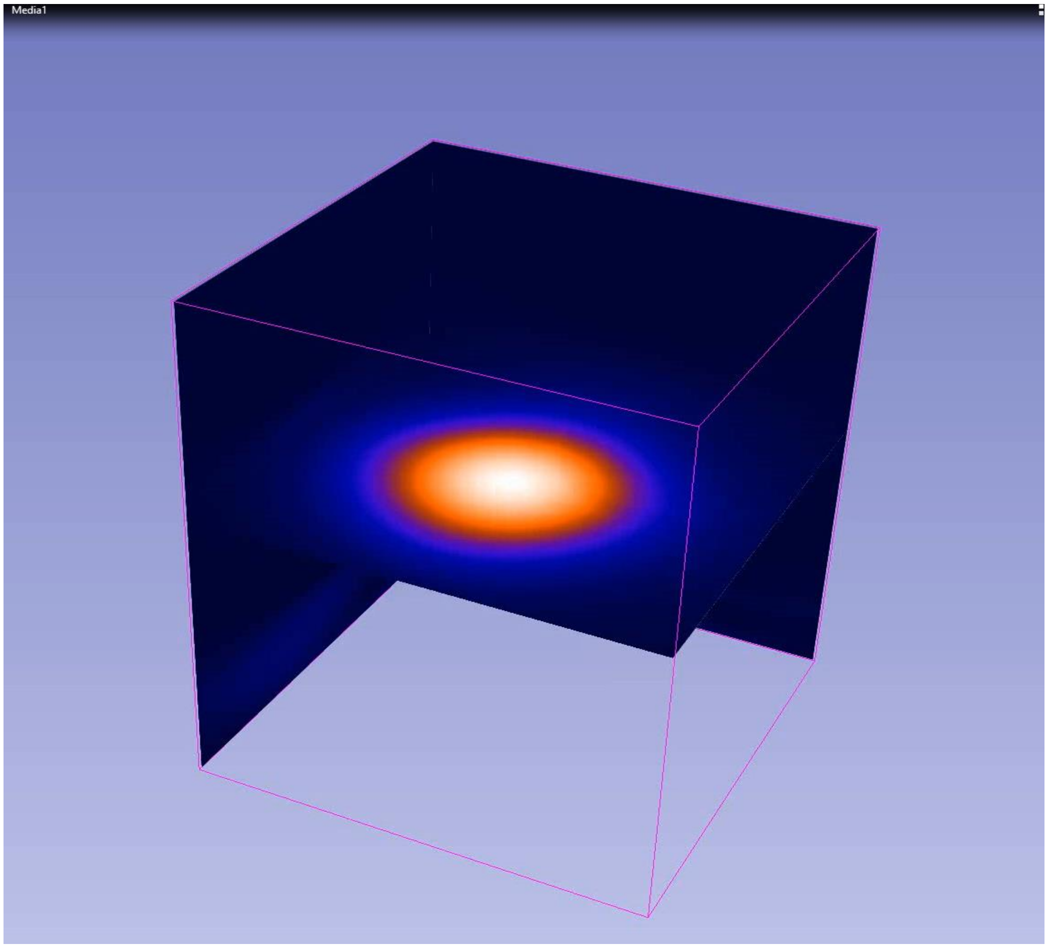

Supplementary video 3 | Real-time tracking of Bragg peak during proton pencil beam scanning with step size of 10 mm

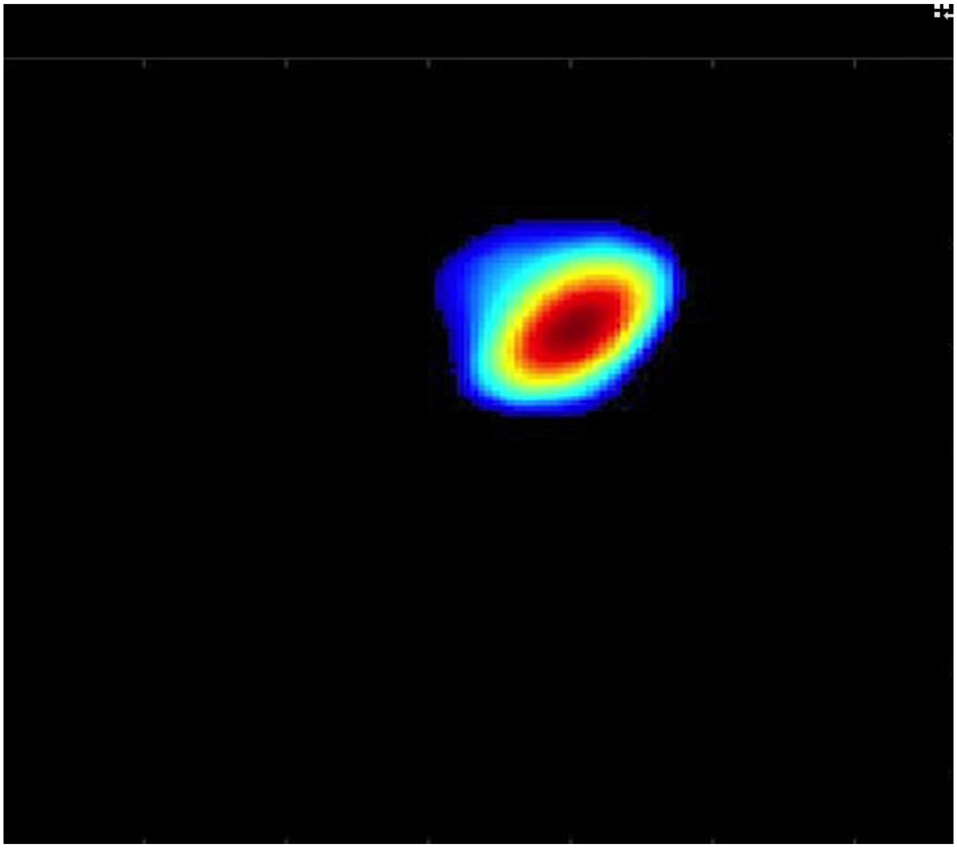

Supplementary video 4 | Real-time tracking of Bragg peak during proton pencil beam scanning with step size of 5 mm

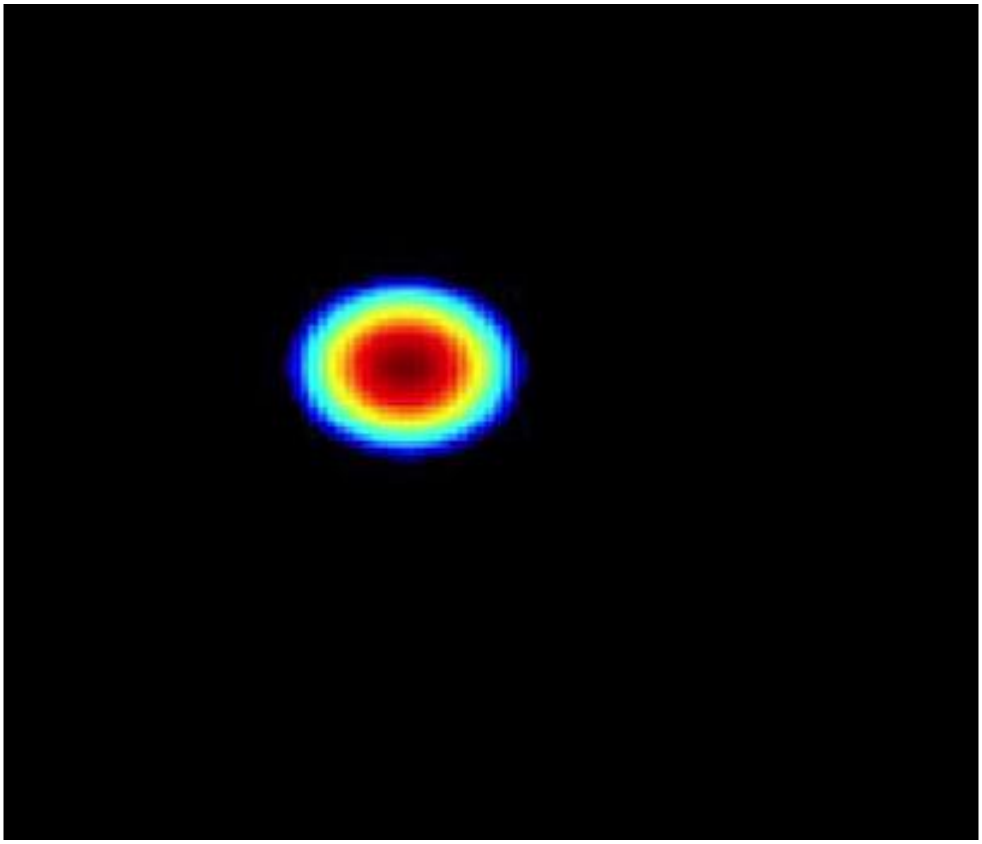

Reference:

1. Baskar, R., Lee, K. A., Yeo, R. & Yeoh, K.-W. Cancer and radiation therapy: current advances and future directions. *Int J Med Sci* **9**, 193–199 (2012).
2. Durante, M., Orecchia, R. & Loeffler, J. S. Charged-particle therapy in cancer: clinical uses and future perspectives. *Nat Rev Clin Oncol* **14**, 483–495 (2017).
3. Chiang, B.-H., Bunker, A., Jin, H., Ahmad, S. & Chen, Y. Developing a Monte Carlo model for MEVION S250i with HYPERSCAN and Adaptive Aperture™ pencil beam scanning proton therapy system. *J Radiother Pract* **20**, 279–286 (2021).
